# Circular RNA mmu_circ_0005019 inhibits fibrosis of cardiac fibroblasts and reverses electrical remodeling of cardiomyocytes

**DOI:** 10.1186/s12872-021-02128-w

**Published:** 2021-06-21

**Authors:** Na Wu, Chengying Li, Bin Xu, Ying Xiang, Xiaoyue Jia, Zhiquan Yuan, Long Wu, Li Zhong, Yafei Li

**Affiliations:** 1 Department of Epidemiology, College of Preventive Medicine, Army Medical University (Third Military Medical University), NO. 30 Gaotanyan Street, Chongqing, 400038 People’s Republic of China; 2 Cardiovascular Disease Center, Third Affiliated Hospital of Chongqing Medical University, Chongqing, 401120 People’s Republic of China

**Keywords:** Atrial fibrillation, Circular RNA, Structural remodeling, Electrical remodeling

## Abstract

**Background:**

Circular RNA (circRNA) have been reported to play important roles in cardiovascular diseases including myocardial infarction and heart failure. However, the role of circRNA in atrial fibrillation (AF) has rarely been investigated. We recently found a circRNA hsa_circ_0099734 was significantly differentially expressed in the AF patients atrial tissues compared to paired control. We aim to investigate the functional role and molecular mechanisms of mmu_circ_0005019 which is the homologous circRNA in mice of hsa_circ_0099734 in AF.

**Methods:**

In order to investigate the effect of mmu_circ_0005019 on the proliferation, migration, differentiation into myofibroblasts and expression of collagen of cardiac fibroblasts, and the effect of mmu_circ_0005019 on the apoptosis and expression of I_to_, I_NA_ and SK3 of cardiomyocytes, gain- and loss-of-function of cell models were established in mice cardiac fibroblasts and HL-1 atrial myocytes. Dual-luciferase reporter assays and RIP were performed to verify the binding effects between mmu_circ_0005019 and its target microRNA (miRNA).

**Results:**

In cardiac fibroblasts, mmu_circ_0005019 showed inhibitory effects on cell proliferation and migration. In cardiomyocytes, overexpression of mmu_circ_0005019 promoted Kcnd1, Scn5a and Kcnn3 expression. Knockdown of mmu_circ_0005019 inhibited the expression of Kcnd1, Kcnd3, Scn5a and Kcnn3. Mechanistically, mmu_circ_0005019 exerted biological functions by acting as a miR-499-5p sponge to regulate the expression of its target gene Kcnn3.

**Conclusions:**

Our findings highlight mmu_circ_0005019 played a protective role in AF development and might serve as an attractive candidate target for AF treatment.

**Supplementary Information:**

The online version contains supplementary material available at 10.1186/s12872-021-02128-w.

## Background

Atrial fibrillation (AF) is the most common arrhythmia. The estimated prevalence is 1–2% in the general population, increasing with the age [1]. It is responsible for an increased risk of strokes, heart failures and all-cause mortality [2]. However, there are clinical dilemma of treatment for AF patients. Antiarrhythmic drugs have only modest efficacy at maintaining sinus rhythm over the long term and are associated with serious side effects [3]. AF relapse following electrical cardioversion is also associated with increased mortality [4]. AF ablation is a well established but imperfect procedure with efficacy varying from 30 to 80% [5]. Therefore, the routine treatment of AF including antiarrhythmic drugs, electrical cardioversion and ablation have limitations.

The upstream therapy which refers to modify the atrial substrate to reduce susceptibility or progression of AF, may be a promising strategy for developing new therapeutic targets [6]. The precise mechanism of AF is the basis of upstream therapy. But the exact mechanism of AF is still unclear. Classical mechanisms which developed since the early twentieth century still form the framework for understanding of the AF pathophysiology. AF maintenance requires an appropriate substrate and a trigger. Ectopic activity can provide the trigger, while structural and electrical remodeling provide the substrate for AF perpetuation [7]. Atrial fibrosis is the hallmark of structural remodeling. In addition, cardiomyocyte apoptosis, increased connective tissue and inflammatory cells infiltration are also present during structural remodeling [8]. The change of some transmembrane ionic currents such as transient outward potassium current (I_to_), inward sodium current (I_NA_) and small conductance calcium-activated potassium channel current (SK) are key determinants of electrical remodeling [9–11]. I_to_ plays a major role in early (phase 1) repolarization. I_NA_ initiates the cardiac action potential. SK current contributes significantly to the repolarization process.

An epigenetic cause may play crucial roles in structural and electrical remodeling. Most of human genome sequences (about 85%) are transcribed into non-coding RNA. Circular RNAs (circRNAs) is a special novel type of non-coding RNA and gained much attention recently [12]. Recent works have suggested that circRNAs may play important roles in the initiation and development of some cardiovascular diseases including atherosclerosis, myocardial infarction and heart failure [13–15]. But the role of circRNA in AF has rarely been investigated. We recently found the expression level of a human circRNA hsa_circ_0099734 (chr13_100368574_100301460_-67114) was significantly higher in the atrial tissues of AF patients compared to paired controls [16]. Therefore, in this study we further investigated the functional role and molecular mechanisms of mice mmu_circ_0005019 in AF which is the homologous circRNA of human hsa_circ_0099734.

## Methods

### Cell culture and treatment

HL-1 atrial myocytes which were derived from the adult mouse atria, was obtained from Zeye Biotech (Shanghai, China), cultured in RPMI-1640 (HyClone, Logan, UT, USA) supplemented with 10% fetal bovine serum (HyClone) and 1% 1X Penicillin–Streptomycin Solution (Beyotime, Shanghai, China). The myocytes were incubated at 37 °C in a 5% CO_2_ atmosphere.

Cardiac fibroblasts were isolated from 10-day-old neonatal mice C57BL/6 (from the animal laboratory center of the Army Medical University). The mice were euthanized by cervical dislocation after sodium pentobarbital anesthesia. The operator had been trained on anesthetized and dead animals. Whole hearts were obtained from neonatal mice, cut into pieces and washed using phosphate-buffered saline (PBS) twice. The tissues was transferred to a tube with 0.08% trypson (HyClone) and incubated at 37 °C for 3 min. The solution containing the cells were collected. The rest of tissue was then placed in 0.08% collagenase II digestion buffer (Gibco, CA, USA) and incubated at 37 °C for 5 min. The digestion/collection process was repeated 3 times. Then the cells were filtered and centrifuged at 1000 rpm, 4 °C for 8 min. Cardiomyocytes were isolated from adherent fibroblasts after incubation for 1.5 h. 0.25% trypson-EDTA and Phenol Red (Gibco, CA, USA) were used in fibroblasts passage. The cardiac fibroblasts were incubated at 37 °C in a 5% CO_2_ atmosphere.

No humans were involved in this study, although the human homologous circRNA of mmu_circ_0005019 was identified by RNA sequencing from the atrial tissues of patients in our previous study [16].

### Plasmid construction and cell transfection

To construct plasmids expressing mmu_circ_0005019, the full-length human mmu_circ_0005019 were synthesized and subcloned into the pcDNA3.1 vector (Invitrogen, CA, USA). The plasmid was verified by Sanger sequencing. The plasmids were transfected using Lipofectamine 2000 Reagent (Invitrogen). For gene knockdown, 100 pmol small interfering RNA (siRNA) were transfected into cells with Lipofectamine 2000 (Invitrogen) in six-well plates. Mmu_circ_0005019 siRNA was designed and synthesized by GenePharma (Shanghai, China), and its sequence is shown in Additional file 1: Table S1. MiR-499-5p, miR-374c-3p and miR-29b-1-5p mimics and inhibitors were purchased from GenePharma (Additional file 1: Table S1).

### Real-time quantitative polymerase chain reaction (qRT-PCR) analysis

Total RNA was isolated from cells using TRIzol reagent (TaKaRa, Dalian, China). For circRNA and mRNA, cDNA was synthesized from total RNA using PrimeScript™ RT reagent Kit with gDNA Eraser (TaKaRa). For miRNA, cDNA was synthesized from total RNA using stem-loop RT-PCR with miRNA RT reagent Kit (Sangon Biotech, Shanghai, China). Real-time PCR was performed using the SYBR Premix Ex Taq (TaKaRa) following the manufacturer's instructions. Housekeeping gene β-actin was used for circRNA and mRNA as an internal standard control. U6 snRNA was used for miRNA as an internal control. All reactions were performed at least in triplicate. Primers sequences used were provided in Additional file 1: Table S2. For the validation of circRNA, The RNase R (Epicenter, CA, USA) digestion reaction was performed following previously published procedures with a ratio of 8U enzyme/8 ng RNA.

### Cell counting kit-8 assay

Cell proliferation was assessed using the cell counting kit-8 (CCK-8, Dojindo Laboratories, Kumamoto, Japan) on days 1, 2, 3, 4 and 5 after transfection. 10 μL of CCK-8 solution was added to each well, the plates were incubated at 37 °C for 2 h, and the absorbance of each well was read at 450 nm using a microplate reader. All of the experiments were performed in triplicate. The cell proliferation curves were plotted using the absorbance at each time point.

### Cell migration

For the migration assays, at 48 h post transfection, 1 × 10^5^ and 5 × 10^4^ cells in serum free media were placed into the upper chamber of an insert (8-μm pore size; Millipore, Billerica, MA, USA) for the effect of mmu_circ_0005019 overexpression and inhibition on cardiac fibroblasts migration, respectively. Medium containing 10% fetal bovine serum was added to the lower chamber. After incubation for 12 h, cells that had migrated through the membrane were stained with methanol and 0.1% crystal violet. All experiments were conducted three times in triplicate.

### Apoptosis analysis

The cell apoptosis was detected by Annexin V-FITC assay kit (Beyotime, Shanghai, China). The cells were centrifuged and 195 μL of Annexin V-FITC binding solution was added. After mixed with 5 μL Annexin V-FITC and 10 μL propidium iodide staining solution, the cells were incubated in an ice bath for 10–20 min, and then for flow cytometry.

### *RNA fluorescence *in situ* hybridization*

Mmu_circ_0005019 head-to-tail probe was synthesized by GenePharma (Shanghai, China). RNA fluorescence in situ hybridization (FISH) was performed using RNA FISH kit (GenePharma) following the manufacturer’s instructions. U6 snRNA and 18S rRNA probes were purchased from GenePharma and were used as nuclear and cytoplasmic localization controls, respectively.

### Luciferase reporter assay

To evaluate the mmu_circ_0005019 and miR-499-5p interaction by luciferase reporter assay, the miR-499-5p binding sites of mmu_circ_0005019 were inserted into the pmirGLO Dual-Luciferase vector (GenePharma). The reconstituted plasmid was named mmu_circ_0005019-WT. The miR-499-5p target site mutations were introduced and inserted into the pmirGLO Dual-Luciferase vector (GenePharma), which was named mmu_circ_0005019-MU. The plasmid construction was performed by GenePharma Corporation. HL-1 cells were seeded into 24-well plates (2 × 10^4^ cells per well) in triplicate for each group. After overnight incubation, cells were co-transfected with reconstituted plasmid and miR-499-5p mimics. Firefly and Renilla luciferase activities were measured 48 h after transfection using the Dual-Luciferase Assay System (Promega, Madison, WI, USA). The relative luciferase activity was calculated using Firefly/Renilla luciferase activity.

### RNA immunoprecipitation (RIP)

RIP was performed using an EZ-Magna RIP Kit (Millipore, Billerica, MA, USA) according to the manufacturer's protocol. HL-1 myocytes were lysed using the RIP lysis buffer. Magnetic beads coupled with anti-immunoglobulin G (IgG) or anti-Argonaute 2 (AGO2) were employed to incubate cell lysates. The expression of mmu_circ_0005019 and miR-499-5p in immunoprecipitated RNAs were assessed by qRT-PCR assay.

### Statistical analysis

To test the differences between two groups, student's t-test was used if the data were normally distributed, otherwise Mann–Whitney U-test was used. All statistical analyses were performed using SPSS statistical software (version 19.0; SPSS Inc., Chicago, IL, USA). A two-sided *P* value < 0.05 was considered to be statistically significant.

The study was carried out in compliance with the ARRIVE guidelines [17].

## Results

### Mmu_circ_0005019 overexpression inhibits cardiac fibroblasts proliferation and migration

**Fig. 1:**
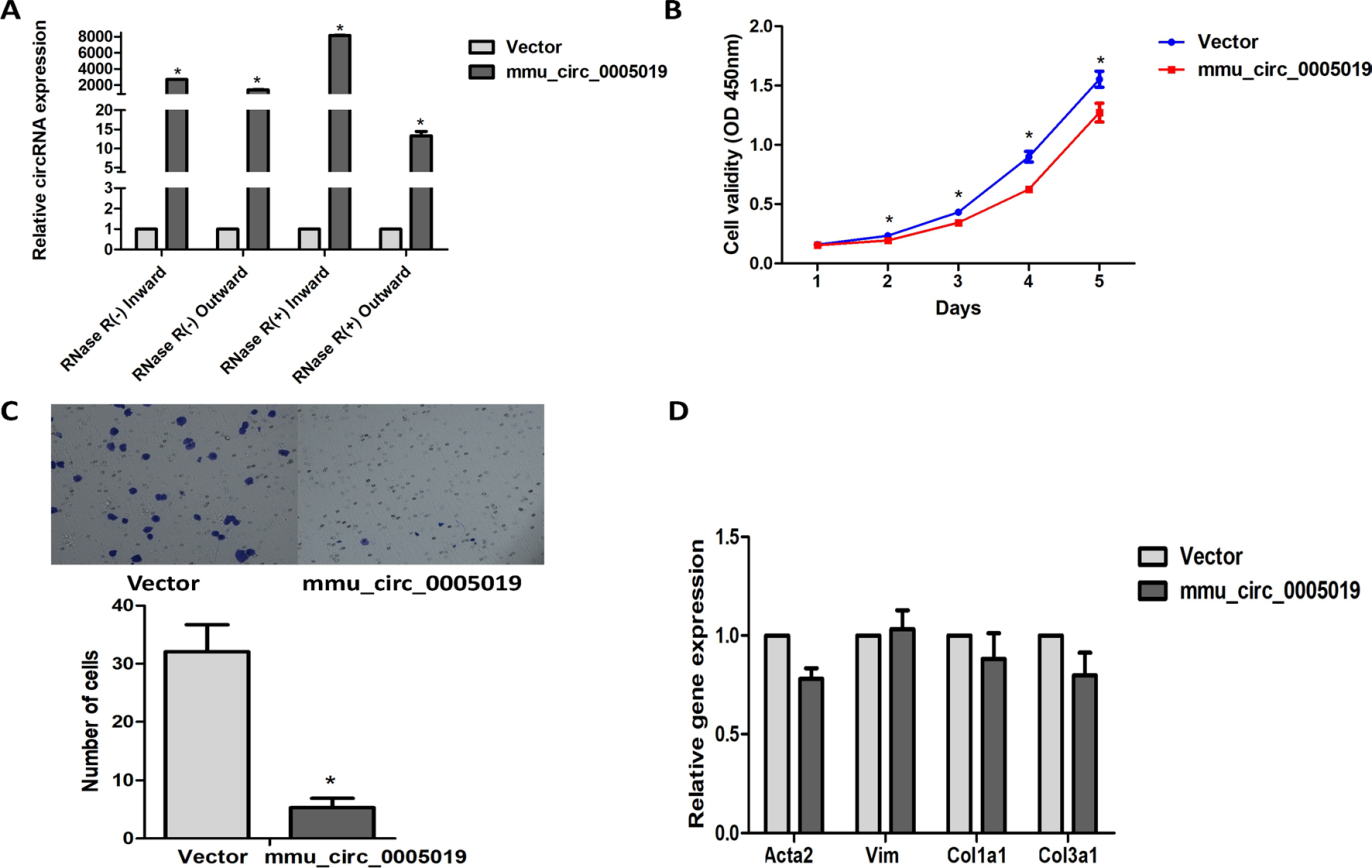
Effect of mmu_circ_0005019 overexpression on the proliferation, migration, differentiation into myofibroblasts and expression of collagen in cardiac fibroblasts. **A** Overexpression of exogenous mmu_circ_0005019 in cardiac fibroblasts were identified by qRT-PCR (Student’s *t*-test). n = 3. **B** Growth curves of cardiac fibroblasts after transfection with mmu_circ_0005019 or vector control were examined by CCK-8 assays (Student’s *t*-test). n = 3. **C** Migration of cardiac fibroblasts transfection with mmu_circ_0005019 or vector control were determined by transwell assays (Mann–Whitney *U* test). n = 10. **D** Relative expression of Acta2, Vim, Cola1 and Col3a1 of cardiac fibroblasts transfection with mmu_circ_0005019 or vector control were determined by qRT-PCR (Student’s *t*-test). n = 3. The results are expressed as the mean ± sem. *mmu_circ_0005019 vs. vector control, *P* < 0.05

To evaluate the functional role of mmu_circ_0005019 in fibrosis, we established gain-of-function cell models by transfecting pcDNA3.1-mmu_circ_0005019 expressing vectors into the cardiac fibroblasts (Fig. 1A). We examined the effects of mmu_circ_0005019 overexpression on cell proliferation, migration, differentiation into myofibroblasts and expression of collagen. CCK-8 assays showed that overexpression of mmu_circ_0005019 significantly inhibited the proliferation of cardiac fibroblasts (Fig. 1B). The transwell assays indicated that overexpression of mmu_circ_0005019 significantly inhibited the migration of cardiac fibroblasts compared with vector control (Fig. 1C). For the role in differentiation into myofibroblasts and expression of collagen, the relative expression of Acta2, Vim, Col1a1 and Col3a1 were determined using qRT-PCR. The test showed that overexpression of mmu_circ_0005019 had no effect on the expression of Acta2, Vim, Col1a1or Col3a1 (Fig. 1D).

### Mmu_circ_0005019 knockdown promotes cardiac fibroblasts migration

**Fig. 2:**
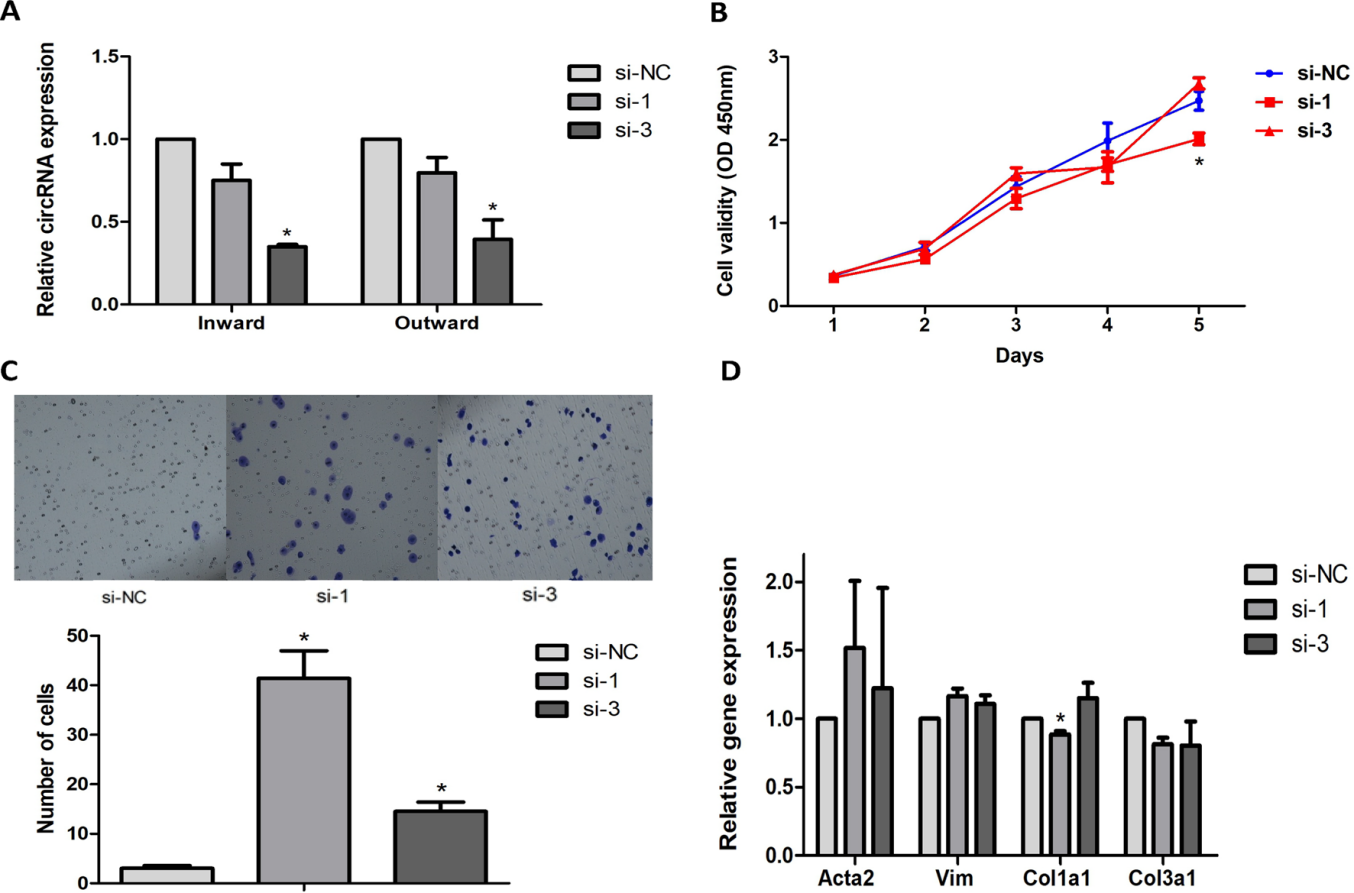
Effect of mmu_circ_0005019 knockdown on the proliferation, migration, differentiation into myofibroblasts and expression of collagen in cardiac fibroblasts. **A** Knockdown of exogenous mmu_circ_0005019 in cardiac fibroblasts were identified by qRT-PCR (Student’s *t*-test). n = 3. **B** Growth curves of cardiac fibroblasts after transfection with mmu_circ_0005019 siRNAs or negative control si-NC were examined by CCK-8 assays (Student’s *t*-test). n = 3. **C** Migration of cardiac fibroblasts transfection with mmu_circ_0005019 siRNAs or negative control si-NC were determined by transwell assays (Mann–Whitney *U* test). n = 10. **D** Relative expression of Acta2, Vim, Cola1 and Col3a1 of cardiac fibroblasts transfection with mmu_circ_0005019 siRNAs or negative control si-NC were determined by qRT-PCR (Student’s *t*-test and Mann–Whitney *U* test). For Col1a1 after si-1 transfection, n = 5; for Acta2 after si-1 transfection, n = 4; for the others, n = 3. The results are expressed as the mean ± sem. *si-1 or si-3 vs. si-NC, *P* < 0.05

We examined the effects of mmu_circ_0005019 knockdown on cell proliferation, migration, differentiation into myofibroblasts and expression of collagen. Two independent single-strand siRNAs were transfected to knockdown the expression of mmu_circ_0005019 (Fig. 2A). CCK-8 assays indicated that knockdown of mmu_circ_0005019 had no effect on the proliferation of cardiac fibroblasts (Fig. 2B). In contrast to the gain-of-function cell models, the transwell assays showed that knockdown of mmu_circ_0005019 significantly promoted the migration of cardiac fibroblasts (Fig. 2C). The relative expression of Col1a1 decreased after knockdown of mmu_circ_0005019. The expression of Acta2, Vim and Col3a1 were not significantly altered (Fig. 2D).

### Mmu_circ_0005019 overexpression promotes expression of Kcnd1, Scn5a and Kcnn3

**Fig. 3:**
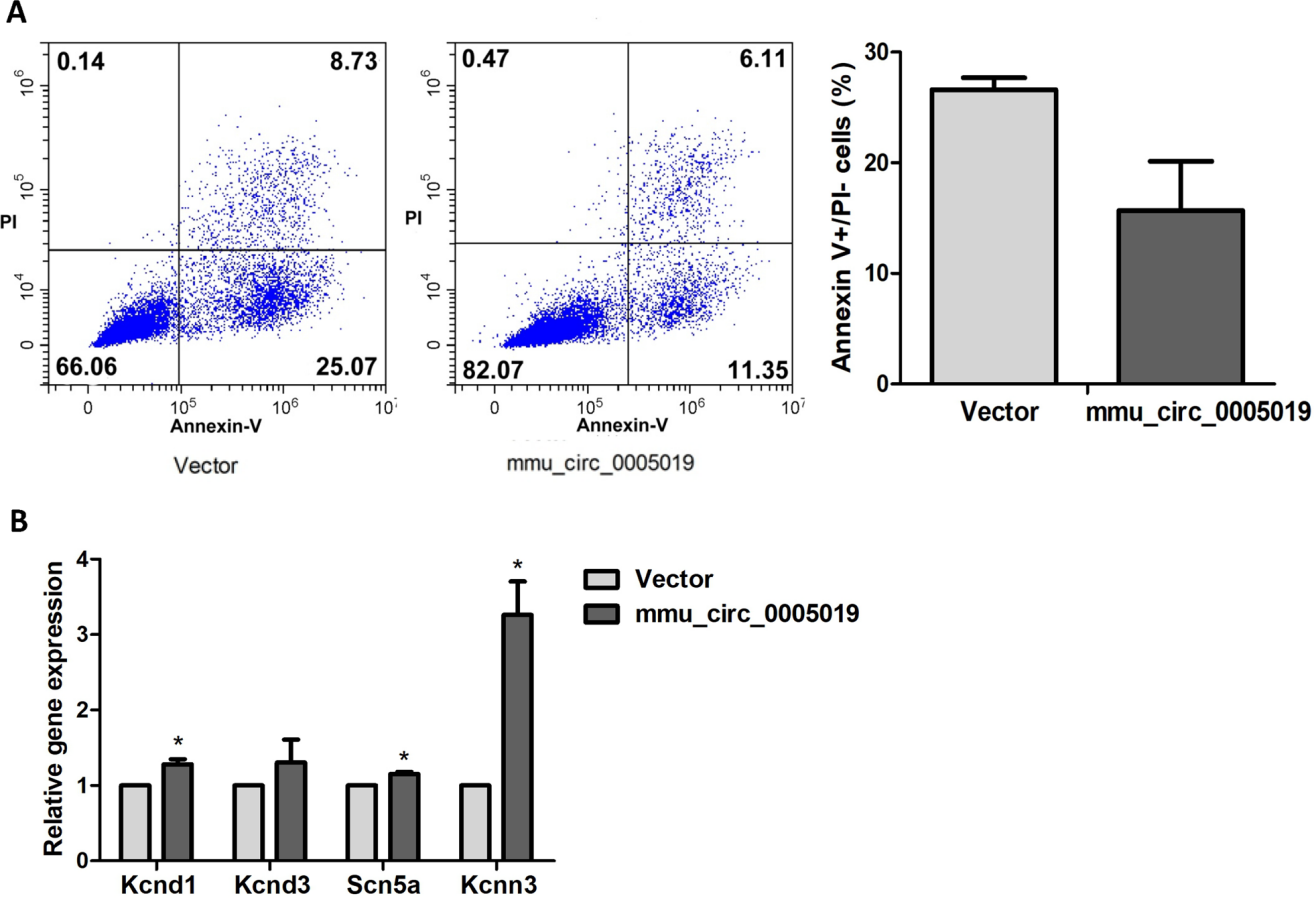
Effect of mmu_circ_0005019 overexpression on the apoptosis and expression of transient outward potassium channel (I_to_), voltage-gated sodium channel (I_NA_) and small conductance calcium-activated potassium channel current 3 (SK3) in cardiomyocytes. **A** Apoptosis of cardiomyocytes transfection with mmu_circ_0005019 or vector control were determined by Annexin V-FITC staining flow cytometry (Mann–Whitney *U* test). n = 3. **B** Relative expression of Kcnd1, Kcnd3, Scn5a and Kcnn3 of cardiomyocytes transfection with mmu_circ_0005019 or vector control were determined by qRT-PCR (Student’s *t*-test and Mann–Whitney *U* test). For Scn5a after mmu_circ_0005019 transfection, n = 5; for the others, n = 3. The results are expressed as the mean ± sem. *mmu_circ_0005019 vs. vector control, *P* < 0.05

To evaluate the functional role of mmu_circ_0005019 in cardiomyocytes, we established gain-of-function cell models by transfecting pcDNA3.1-mmu_circ_0005019 expressing vectors into the HL-1 cardiomyocytes. Annexin V-FITC assay showed that overexpression of mmu_circ_0005019 decreased the cardiomyocytes apoptosis, but the change was not significant (Fig. 3A).

Kcnd1 and Kcnd3 encoded I_to_. Scn5a and Kcnn3 encoded I_NA_ and SK3, respectively. The relative expression of Kcnd1, Kcnd3, Scn5a and Kcnn3 increased after overexpression of mmu_circ_0005019, but only the increase of Kcnd1, Scn5a and Kcnn3 were significant (Fig. 3B).

### Mmu_circ_0005019 knockdown inhibits expression of Kcnd1, Kcnd3, Scn5a and Kcnn3

**Fig. 4:**
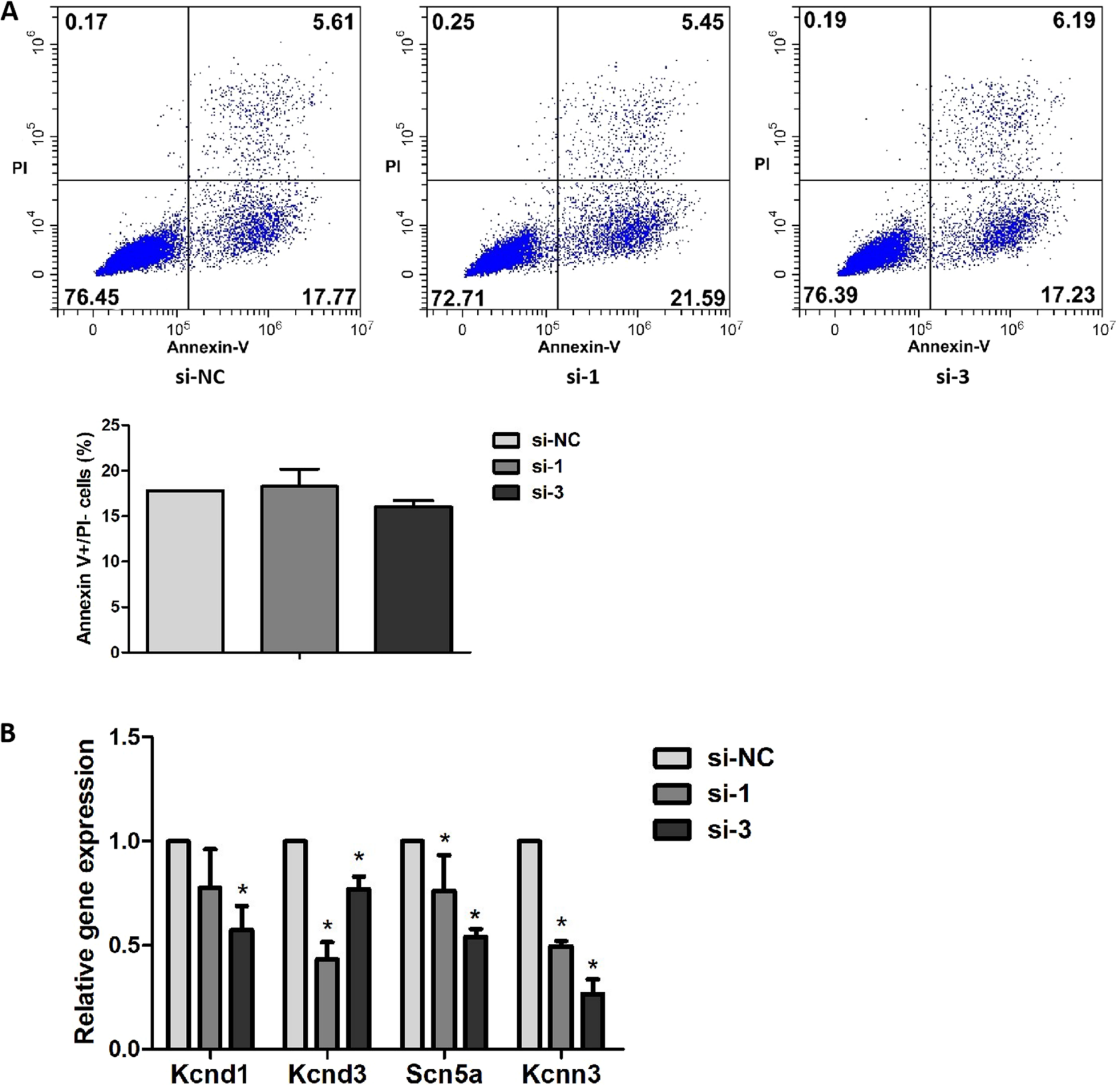
Effect of mmu_circ_0005019 knockdown on the apoptosis and expression of transient outward potassium channel (I_to_), voltage-gated sodium channel (I_NA_) and small conductance calcium-activated potassium channel current 3 (SK3) in cardiomyocytes. **A** Apoptosis of cardiomyocytes transfection with mmu_circ_0005019 siRNAs or negative control si-NC were determined by Annexin V-FITC staining flow cytometry (Student’s *t*-test). n = 3. **B** Relative expression of Kcnd1, Kcnd3, Scn5a and Kcnn3 of cardiomyocytes transfection with mmu_circ_0005019 siRNAs or negative control si-NC were determined by qRT-PCR (Student’s *t*-test and Mann–Whitney *U* test). For Kcnd1 after si-1 transfection, n = 6; for Kcnd3 and Scn5a after si-1 transfection, n = 4; for the others, n = 3. The results are expressed as the mean ± sem. *si-1 or si-3 vs. si-NC, *P* < 0.05

Knockdown of mmu_circ_0005019 had no effect on the cardiomyocytes apoptosis after two independent siRNAs transfection (Fig. 4A). The relative expression of Kcnd1, Kcnd3, Scn5a and Kcnn3 significantly decreased after knockdown of mmu_circ_0005019 (Fig. 4B).

### Mmu_circ_0005019 serves as a sponge for miR-499-5p by targeting Kcnn3

**Fig. 5:**
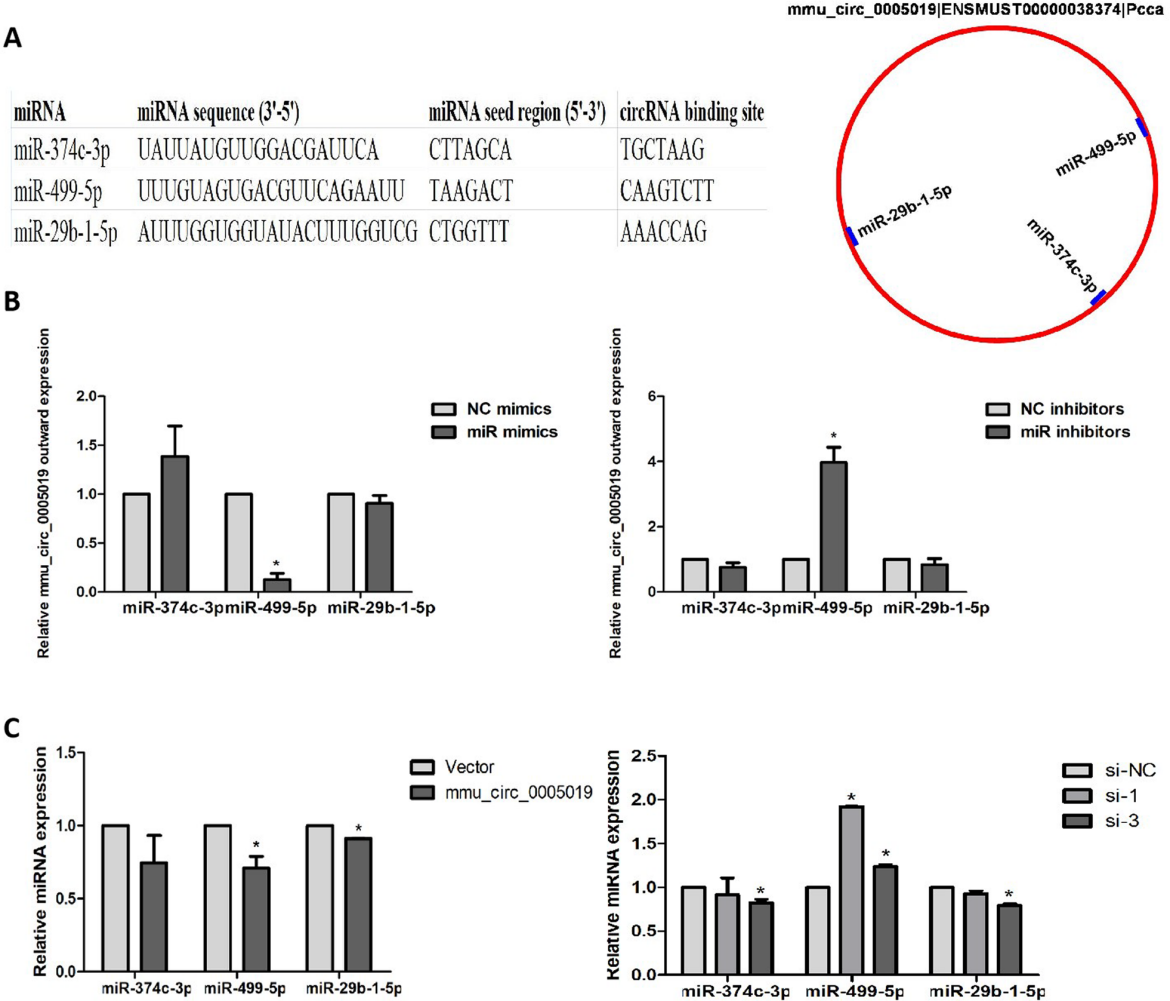
mmu_circ_0005019 target candidate miRNAs. **A** MiRanda algorithm was used to predict which miRNAs might have binding sites with mmu_circ_0005019. **B** Mmu_circ_0005019 expression levels in cardiomyocytes after transfection with miR-374c-3p, miR-499-5p and miR-29b-1-5p mimics or inhibitors (Student’s *t*-test). For miR-29b-1-5p mimics transfection, n = 6; for miR-374c-3p and miR-29b-1-5p inhibitors transfection, n = 5; for the others, n = 3. **C** miR-374c-3p, miR-499-5p and miR-29b-1-5p expression levels in cardiomyocytes after overexpression or knockdown of mmu_circ_0005019 (Student’s *t*-test). n = 3. The results are expressed as the mean ± sem. *P < 0.05

**Fig. 6:**
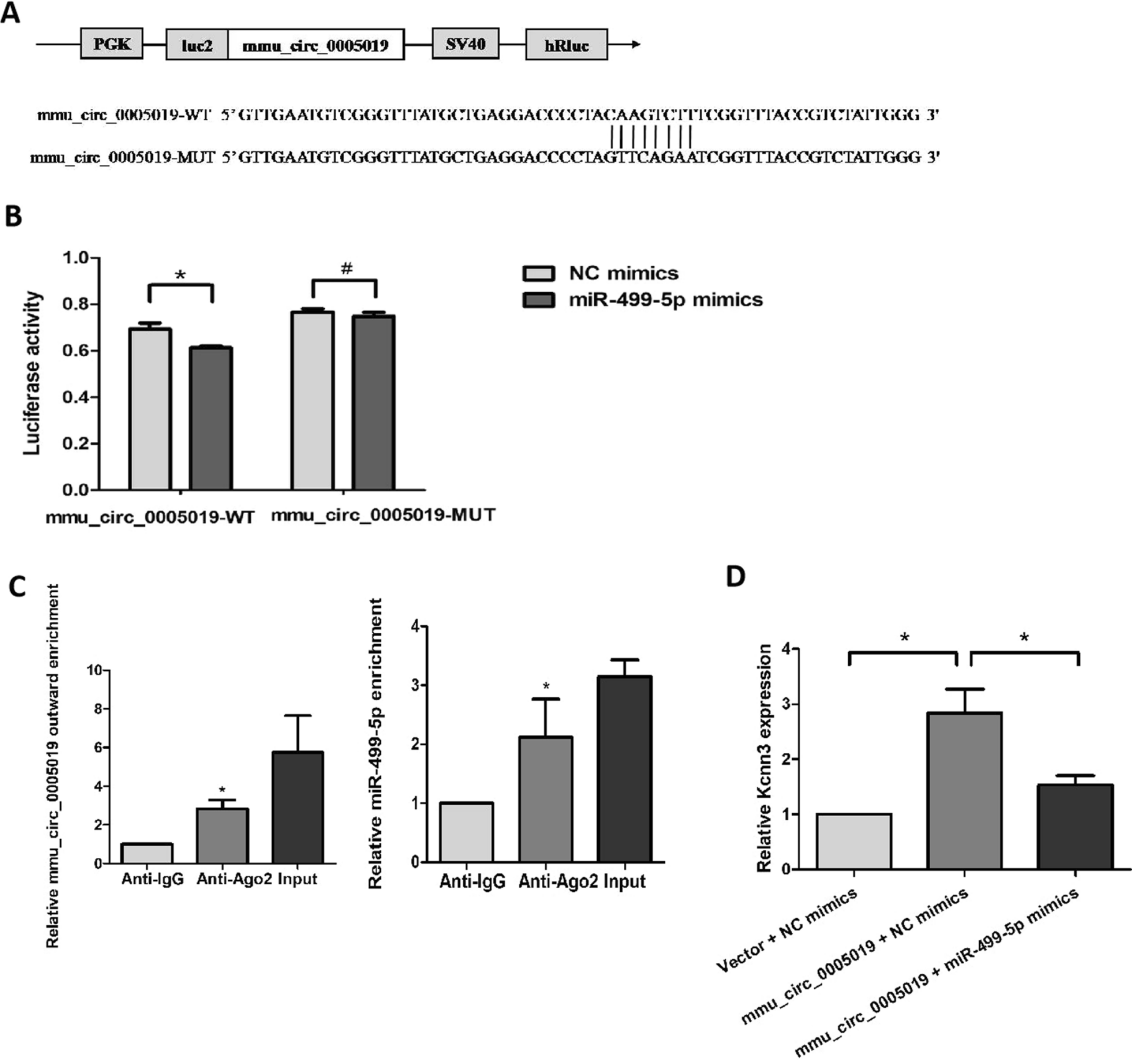
mmu_circ_0005019 functions as a sponge for miR-499-5p. **A** Schematic of mmu_circ_0005019-WT (wild-type) and mmu_circ_0005019-MUT (mutant) pmirGLO Dual-Luciferase luciferase vectors. **B** Luciferase assays of cardiomyocytes cells co-transfected with mmu_circ_0005019-WT or mmu_circ_0005019-MUT reporter and miR-499-5p mimics or negative control (NC) mimics. n = 3. *Student’s *t*-test, *P* < 0.05; # Student’s *t*-test, *P* > 0.05. **C** mmu_circ_0005019 and miR-499-5p levels were determined by qRT-PCR after Ago2 or IgG RIP assay. n = 3. *Student’s *t*-test or Mann–Whitney *U* test, *P* < 0.05. **D** Relative expression of Kcnn3 of cardiomyocytes after co-transfection with mmu_circ_0005019 or control vector and miR-499-5p mimics or NC mimics. n = 3. * Student’s *t*-test, *P* < 0.05. The results are expressed as the mean ± sem

We next investigated the potential molecular mechanisms of mmu_circ_0005019 regulating Kcnn3 expression in cardiomyocytes. To evaluate whether mmu_circ_0005019 might function as natural miRNA sponges to prevent miRNA from binding their target mRNA, we first screened the possible target candidate miRNAs. We selected miRNAs which were negatively correlated with mmu_circ_0005019 expression in our previous miRNA and circRNA databases of RNA sequencing [16], and were predicted to target mmu_circ_0005019 using the miRanda algorithm. Then miR-374c-3p was selected according to this criteria. In addition, we selected miRNAs which were reported to associated with AF in previous studies and were predicted to target mmu_circ_0005019 using the miRanda algorithm. Then miR-499-5p and miR-29b-1-5p were selected (Fig. 5A) [18, 19]. Subsequently, we investigated the expression levels of mmu_circ_0005019 after knockdown or overexpression of miR-374c-3p, miR-499-5p and miR-29b-1-5p. Among these three miRNAs, only the transfection of miR-499-5p inhibitors significantly increased mmu_circ_0005019 expression levels, whereas the miR-499-5p mimics significantly down-regulated mmu_circ_0005019 in HL-1 myocytes (Fig. 5B). We then detected the expression of miR-374c-3p, miR-499-5p and miR-29b-1-5p in HL-1 cardiomyocytes with mmu_circ_0005019 overexpression or knockdown. And we found that after overexpression or knockdown of mmu_circ_0005019 in HL-1 cardiomyocytes, only miR-499-5p was significantly down-regulated or upregulated, respectively (Fig. 5C). For further confirmation, a luciferase reporter assay was performed and the results showed that overexpression of miR-499-5p significantly reduced the luciferase activities of the mmu_circ_0005019-WT reporter vector but not the mmu_circ_0005019-MUT reporter vector (Fig. 6A, B). In addition, RIP assay demonstrated that mmu_circ_0005019 and miR-499-5p were significantly enriched in the Ago2 IP fraction in comparison to the IgG control fractions (Fig. 6C). Functionally, mmu_circ_0005019 could promote the expression of Kcnn3, and overexpression of miR-499-5p partially rescue the effect of mmu_circ_0005019 on Kcnn3 in HL-1 cardiomyocytes (Fig. 6D). Kcnn3 has been validated to be miR-499-5p target gene in a previous study [18]. These results implied that mmu_circ_0005019 serves as a miR-499-5p sponge to regulate the expression of its target gene Kcnn3.

## Discussion

In this study, we found that mmu_circ_0005019, the homologous circRNA in mice of hsa_circ_0099734, could inhibit cardiac fibroblasts proliferation and migration. It also could promote the expression of Kcnd1, Scn5a and Kcnn3 which encoded I_to_, I_NA_ and SK3, respectively in vitro. Mechanistically, mmu_circ_0005019 exerted biological functions by acting as a miR-499-5p sponge to regulate the expression of its target gene Kcnn3. Therefore, it might play a protective role in AF development. The exact mechanisms underlying AF remains unclear. But it is well recognized that structural and electrical remodeling provide the substrate for AF perpetuation. Fibrosis is a hallmark of arrhythmogenic structural remodeling, and cardiac fibroblasts play crucial roles in fibrosis due to their ability to synthesize and breakdown the extracellular matrix (ECM) [20]. Tissue fibrosis results from an accumulation of fibrillar collagen (mainly types I and III) which is mainly produced by cardiac fibroblasts [21]. Myofibroblasts which are not part of normal cardiac tissue but differentiate from cardiac fibroblasts also play important role in fibrosis. Their ability to synthesize collagen is stronger than that of cardiac fibroblasts, and are highly responsive to chemokines released [22]. For detection of myofibroblast differentiation, α-smooth muscle actin (α-SMA; gene ACTA) and vimentin (gene VIM) expression are common key elements [23].

In this study, we found that mmu_circ_0005019 could inhibit cardiac fibroblasts proliferation and migration in vivo, indicating that mmu_circ_0005019 could inhibit fibrosis. However, both overexpression and knockdown of mmu_circ_0005019 had little effect on Col1a1or Col3a1 which encode collagen types I and III, respectively. Therefore, the role mmu_circ_0005019 plays in inhibiting fibrosis is limited. Several studies have explored the functional role circRNAs in fibrosis. CircRNA_000203 could enhance the expressions of Col1a2, Col3a1 and α-SMA in cardiac fibroblasts. Mechanically, circRNA_000203 sponged miR-26b-5p to derepress the downstream targets of Col1a2 and CTGF [24]. CircRNA_010567 was found to promote myocardial fibrosis via suppressing miR-141 by targeting TGF-beta1 [25]. CircNFIB could attenuate cardiac fibrosis by sponging miR-433 [26].

Electrical remodeling involving ionic channel is another important basis for AF initiation and maintenance. The transient outward current (I_to_) plays a major role in early (phase 1) repolarization. The heterologous expression of K^+^ channel α-subunits, including Kv4.3 and Kv4.1 results in rapidly activating and inactivating K^+^ currents [10]. I_to_ current, Kcnd3 and Kcnd1 mRNA expression decreased in AF [9]. The voltage-gated cardiac sodium channel, SCN5A, conducts the inward sodium current (I_NA_) that initiates the cardiac action potential. Mutations or rare variants in SCN5A may predispose patients with AF. I_NA_ current density and SCN5A expression reduced in AF [27, 28]. KCNN3 encodes the small conductance calcium-activated potassium channel 3 (SK3). The SK channel family consists of three members, SK1 (K_Ca_2.1), SK2 (K_Ca_2.2), and SK3 (K_Ca_2.3) encoded by three distinct genes, KCNN1, KCNN2, and KCNN3. SK current contributes significantly to the repolarization process. It has been revealed that different channel subunits can heteromultimerize, and genetic variation in one isoform may affect the function of other isoforms and the overall SK current [11]. Genome-wide association studies (GWAS) identified KCNN3 was strongly associated with AF [29, 30]. The gene-targeted animals overexpress the SK3 channel show a significant shortening of the action potential duration (APD). Conversely, treatment with dietary doxycycline results in a significant prolongation of APD [31].

In this study, we found that overexpression of mmu_circ_0005019 significantly increased Kcnd1, Scn5a and Kcnn3 expression level, while knockdown of mmu_circ_0005019 significantly decreased Kcnd1, Kcnd3, Scn5a and Kcnn3. Since previous studies showed that decreased Kcnd1, Kcnd3, Scn5a and Kcnn3 expression levels were associated with AF [9, 18, 32], our results indicated that mmu_circ_0005019 might inhibit electrical remodeling in vivo. To our knowledge, there were no studies investigating the association of circRNA with genes encoding ionic channels. Jiang et al. predicted that circRNA_100612 might regulate the expression of KCNIP1 and JPH2 through binding miR-133b according to circRNA microarray [33]. But no experiments were performed to validate this proposal.

Increased cardiomyocytes apoptosis were observed in the atrial tissues of AF patients and animal models [34]. Several studies have reported that some circRNAs could promote cardiomyocytes apoptosis including circRNA MFACR and circNCX1 [35, 36], while some could inhibit apoptosis including circ-Ttc3 [37]. In this study, overexpression or knockdown of mmu_circ_0005019 had no effect on apoptosis.

It has been elucidated that circRNAs can function as miRNA sponges, as target-RNA decoys by binding to RBPs, as regulators of splicing and transcription by binding snRNA and enhancing Pol II activities, and as protein scaffolds and modifiers of parental gene expression [38]. At present, the most widely studied function is competing endogenous RNAs or miRNA sponges. Therefore, we firstly explored that whether mmu_circ_0005019 could function as a natural miRNA sponge to regulate Kcnn3 expression. We screened miRNAs which were reported to be associated with AF in previous studies and predicted to target mmu_circ_0005019, then miR-499-5p was selected. A previous study has verified experimentally that miR-499 binds to the 3’UTR of KCNN3 mRNA, and could regulated KCNN3 expression [18]. In this study, according to luciferase reporter and RIP assay we found that mmu_circ_0005019 could function as a natural sponge for miR-499-5p, regulating the expression of the miR-499-5p target gene, Kcnn3. Overexpression of miR-449-5p could partially rescue the promoting effect of mmu_circ_0005019 on Kcnn3. Since previous studies showed that decreased KCNN3 expression levels were associated with AF [18], our results indicated that mmu_circ_0005019 might inhibit electrical remodeling in vitro*,* which was consistent with the role mmu_circ_0005019 played in regulating Kcnd1, Kcnd3 and Scn5a. But further studies are needed to explore the molecular mechanisms of that mmu_circ_0005019 inhibited fibroblasts proliferation, migration, expression of collagen, cardiomyocytes apoptosis, and altered expression of Kcnd1, Kcnd3 and Scn5a.

How hsa_circ_0099734 was expressed in different stages of AF development and progression remained unknown. In our previous study, we found that the expression level of hsa_circ_0099734 was significantly higher in the atrial tissues of AF patients compared to paired controls [16]. The increased level of hsa_circ_0099734 in AF might due to a compensative mechanism. Structural and electrical remodeling triggers a redistribution of hsa_circ_0099734 in atria. These findings indicated that hsa_circ_0099734 was necessary to maintain a correct balance of cardiac function.

Several limitations of this study should be considered when interpreting our results. Firstly, although mmu_circ_0005019 is the homologous circRNA in mice of hsa_circ_0099734, we did not validate the functional role and mechanism of hsa_circ_0099734 in human cardiomyocytes, and the external validity of this study is limited. Secondly, the protein expressions were not evaluated by western blot. Finally, we did not perform experiments on ionic currents, action potential, intracellula/extracellular ion density to investigate the electrical remodeling of cardiomyocytes comprehensively.

## Conclusions

This study demonstrates that mmu_circ_0005019 which is the mice homologous circRNA of has_circ_0099734 could inhibit fibrosis and reverse electrical remodeling of I_to_, I_NA_ and SK3. Mmu_circ_0005019 could function as a natural sponge for miR-499-5p, and regulate the expression of the miR-499-5p target gene, Kcnn3.

These findings provide a basis for further understanding of the aetiology underlying. It is of great significance to find new therapeutic targets, improve the diagnosis and prognosis of AF.

## Supplementary Information

**Additional file 1**. Supplementary Information.

## Data Availability

The datasets generated and analyzed during the current study are available from the corresponding author on reasonable request.

## Abbreviations

AF: Atrial fibrillation
I_to_: Transient outward potassium current
I_NA_: Voltage-gated sodium channels current
SK: Small conductance calcium-activated potassium channel
PBS: Phosphate-buffered saline
RIP: RNA immunoprecipitation
ECM: Extracellular matrix
α-SMA: α-Smooth muscle actin
GWAS: Genome-wide association studies
APD: Action potential duration

## References

[1] January CT, Wann LS, Calkins H, Chen LY, Cigarroa JE, Cleveland JC (2019). 2019 AHA/ACC/HRS focused update of the 2014 AHA/ACC/HRS guideline for the management of patients with atrial fibrillation: a report of the American College of Cardiology/American Heart Association Task Force on Clinical Practice Guidelines and the Heart Rhythm Society. J Am Coll Cardiol.

[2] Passman R, Bernstein RA (2016). New appraisal of atrial fibrillation Burden and Stroke prevention. Stroke.

[3] Calvo D, Filgueiras-Rama D, Jalife J (2018). Mechanisms and drug development in atrial fibrillation. Pharmacol Rev.

[4] Ecker V, Knoery C, Rushworth G, Rudd I, Ortner A, Begley D (2018). A review of factors associated with maintenance of sinus rhythm after elective electrical cardioversion for atrial fibrillation. Clin Cardiol.

[5] Verma A, Macle L (2018). Persistent atrial fibrillation ablation: where do we go from here?. Can J Cardiol.

[6] Smit MD, Van Gelder IC (2009). Upstream therapy of atrial fibrillation. Expert Rev Cardiovasc Ther.

[7] Iwasaki YK, Nishida K, Kato T, Nattel S (2011). Atrial fibrillation pathophysiology: implications for management. Circulation.

[8] Dzeshka MS, Lip GY, Snezhitskiy V, Shantsila E (2015). Cardiac fibrosis in patients with atrial fibrillation: mechanisms and clinical implications. J Am Coll Cardiol.

[9] Caballero R, de la Fuente MG, Gomez R, Barana A, Amoros I, Dolz-Gaiton P (2010). In humans, chronic atrial fibrillation decreases the transient outward current and ultrarapid component of the delayed rectifier current differentially on each atria and increases the slow component of the delayed rectifier current in both. J Am Coll Cardiol.

[10] Wang Z, Feng J, Shi H, Pond A, Nerbonne JM, Nattel S (1999). Potential molecular basis of different physiological properties of the transient outward K+ current in rabbit and human atrial myocytes. Circ Res.

[11] Tuteja D, Xu D, Timofeyev V, Lu L, Sharma D, Zhang Z (2005). Differential expression of small-conductance Ca2+-activated K+ channels SK1, SK2, and SK3 in mouse atrial and ventricular myocytes. Am J Physiol Heart Circ Physiol.

[12] Hansen TB, Jensen TI, Clausen BH, Bramsen JB, Finsen B, Damgaard CK (2013). Natural RNA circles function as efficient microRNA sponges. Nature.

[13] Burd CE, Jeck WR, Liu Y, Sanoff HK, Wang Z, Sharpless NE (2010). Expression of linear and novel circular forms of an INK4/ARF-associated non-coding RNA correlates with atherosclerosis risk. PLoS Genet.

[14] Geng HH, Li R, Su YM, Xiao J, Pan M, Cai XX (2016). The circular RNA Cdr1as promotes myocardial infarction by mediating the regulation of miR-7a on its target genes expression. PLoS ONE.

[15] Wang K, Long B, Liu F, Wang JX, Liu CY, Zhao B (2016). A circular RNA protects the heart from pathological hypertrophy and heart failure by targeting miR-223. Eur Heart J.

[16] Wu N, Li J, Chen XH, Xiang Y, Wu L, Li CY (2020). Identification of long non-coding RNA and circular RNA expression profiles in atrial fibrillation. Heart Lung Circ.

[17] du Sert NP, Hurst V, Ahluwalia A, Alam S, Avey MT, Baker M, et al. The ARRIVE guidelines 2.0: Updated guidelines for reporting animal research. PLoS Biol. 2020; 18(7). 10.1371/journal.pbio.3000410 PMC7360023 32663219

[18] Ling TY, Wang XL, Chai Q, Lau TW, Koestler CM, Park SJ (2013). Regulation of the SK3 channel by microRNA-499–potential role in atrial fibrillation. Heart Rhythm.

[19] van Rooij E, Sutherland LB, Thatcher JE, DiMaio JM, Naseem RH, Marshall WS (2008). Dysregulation of microRNAs after myocardial infarction reveals a role of miR-29 in cardiac fibrosis. Proc Natl Acad Sci U S A.

[20] Lajiness JD, Conway SJ (2012). The dynamic role of cardiac fibroblasts in development and disease. J Cardiovasc Transl Res.

[21] Zeisberg EM, Kalluri R (2010). Origins of cardiac fibroblasts. Circ Res.

[22] Baum J, Duffy HS (2011). Fibroblasts and myofibroblasts: what are we talking about?. J Cardiovasc Pharmacol.

[23] Hinz B, Phan SH, Thannickal VJ, Prunotto M, Desmouliere A, Varga J (2012). Recent developments in myofibroblast biology paradigms for connective tissue remodeling. Am J Pathol.

[24] Tang CM, Zhang M, Huang L, Hu ZQ, Zhu JN, Xiao Z (2017). CircRNA_000203 enhances the expression of fibrosis-associated genes by derepressing targets of miR-26b-5p, Col1a2 and CTGF, in cardiac fibroblasts. Sci Rep.

[25] Zhou B, Yu JW (2017). A novel identified circular RNA, circRNA_010567, promotes myocardial fibrosis via suppressing miR-141 by targeting TGF-beta1. Biochem Biophys Res Commun.

[26] Zhu Y, Pan W, Yang T, Meng X, Jiang Z, Tao L (2019). Upregulation of circular RNA CircNFIB attenuates cardiac fibrosis by sponging miR-433. Front Genet.

[27] Darbar D, Kannankeril PJ, Donahue BS, Kucera G, Stubblefield T, Haines JL (2008). Cardiac sodium channel (SCN5A) variants associated with atrial fibrillation. Circulation.

[28] Ellinor PT, Nam EG, Shea MA, Milan DJ, Ruskin JN, MacRae CA (2008). Cardiac sodium channel mutation in atrial fibrillation. Heart Rhythm.

[29] Ellinor PT, Lunetta KL, Glazer NL, Pfeufer A, Alonso A, Chung MK (2010). Common variants in KCNN3 are associated with lone atrial fibrillation. Nat Genet.

[30] Olesen MS, Jabbari J, Holst AG, Nielsen JB, Steinbruchel DA, Jespersen T (2011). Screening of KCNN3 in patients with early-onset lone atrial fibrillation. Europace.

[31] Zhang XD, Timofeyev V, Li N, Myers RE, Zhang DM, Singapuri A (2014). Critical roles of a small conductance Ca(2)(+)-activated K(+) channel (SK3) in the repolarization process of atrial myocytes. Cardiovasc Res.

[32] Gaspo R, Bosch RF, Bou-Abboud E, Nattel S (1997). Tachycardia-induced changes in Na+ current in a chronic dog model of atrial fibrillation. Circ Res.

[33] Jiang S, Guo C, Zhang W, Che W, Zhang J, Zhuang S (2019). The integrative regulatory network of circRNA, microRNA, and mRNA in atrial fibrillation. Front Genet.

[34] Tsoporis JN, Fazio A, Rizos IK, Izhar S, Proteau G, Salpeas V (2018). Increased right atrial appendage apoptosis is associated with differential regulation of candidate MicroRNAs 1 and 133A in patients who developed atrial fibrillation after cardiac surgery. J Mol Cell Cardiol.

[35] Wang K, Gan TY, Li N, Liu CY, Zhou LY, Gao JN (2017). Circular RNA mediates cardiomyocyte death via miRNA-dependent upregulation of MTP18 expression. Cell Death Differ.

[36] Li M, Ding W, Tariq MA, Chang W, Zhang X, Xu W (2018). A circular transcript of ncx1 gene mediates ischemic myocardial injury by targeting miR-133a-3p. Theranostics.

[37] Cai L, Qi B, Wu X, Peng S, Zhou G, Wei Y (2019). Circular RNA Ttc3 regulates cardiac function after myocardial infarction by sponging miR-15b. J Mol Cell Cardiol.

[38] Memczak S, Jens M, Elefsinioti A, Torti F, Krueger J, Rybak A (2013). Circular RNAs are a large class of animal RNAs with regulatory potency. Nature.

