# Supplementary material for: Circular RNA mmu_circ_0005019 inhibits fibrosis of cardiac fibroblasts and reverses electrical remodeling of cardiomyocytes
Source: BMC Cardiovasc Disord. 2021 Jun 21;21:308. doi: 10.1186/s12872-021-02128-w (PMC8215745; doi:10.1186/s12872-021-02128-w)
Supplement: Supplementary file 1 — Additional file 1. Supplementary Information. [file 12872_2021_2128_MOESM1_ESM.docx]

Supplementary Material

Circular RNA mmu_circ_0005019 inhibits fibrosis of cardiac fibroblasts and reverses electrical remodeling of cardiomyocytes

Na Wu^1 †^, Chengying Li^1†^, Bin Xu^1^, Ying Xiang^1^ , Xiaoyue Jia^1^, Zhiquan Yuan^1^, Long Wu^1^ , Li Zhong^2 *^, Yafei Li^1*^

^1^ Department of Epidemiology, College of Preventive Medicine, Army Medical University (Third Military Medical University), Chongqing 400038, People’s Republic of China

^2^ Cardiovascular Disease Center, Third Affiliated Hospital of Chongqing Medical University, Chongqing 401120, People’s Republic of China

^†^ These authors contributed equally to this work.

^*^ These authors jointly directed the project.

Correspondence:

Yafei Li, Ph.D.

Department of Epidemiology, College of Preventive Medicine, Army Medical University (Third Military Medical University), NO.30 Gaotanyan Street, Chongqing 400038, People’s Republic of China

E-mail: liyafei2008@hotmail.com; liyafei2008@tmmu.edu.cn;

Telephone: +86 23 68771535

Supplementary Table S1. Sequences of mmu_circ_0005019 siRNAs, mimics and inhibitors

Supplementary Table S2. Primers used in this study

Supplementary Table S1. Sequences of mmu_circ_0005019 siRNAs, mimics and inhibitors

| Gene | Sense (5’-3’) |
| --- | --- |
| siRNA-1 (si-1) | GCCGGCAUUUACCGUCGAATT |
| siRNA-3 (si-3) | CCGGCAUUUACCGUCGAACTT |
| siRNA-NC (si-NC) | UUCUCCGAACGUGUCACGUTT |
| mmu-miR-499-5p mimic | UUAAGACUUGCAGUGAUGUUU |
| mmu-miR-374c-3p mimic | ACUUAGCAGGUUGUAUUAU |
| mmu-miR-29b-1-5p mimic | GCUGGUUUCAUAUGGUGGUUUA |
| mmu-miR-499-5p inhibitor | AAACAUCACUGCAAGUCUUAA |
| mmu-miR-374c-3p inhibitor | AUAAUACAACCUGCUAAGU |
| mmu-miR-29b-1-5p inhibitor | GCUGGUUUCAUAUGGUGGUUUA |

Supplementary Table S2. Primers used in this study

| Gene | Sense (5’-3’) |
| --- | --- |
| mmu_circ_0005019 Inward | Forward: TTACCCACTCAGGCACAAGC |
|  | Reverse: GGACAGCCTCCCAATAGACG |
| mmu_circ_0005019 Outward | Forward: AGGGCACACGTTAACACTGA |
|  | Reverse: AGTCCAGGCCAGTAATGCAC |
| Acta2 | Forward: GCCCAGAGCAAGAGAGG |
|  | Reverse: TGTCAGCAGTGTCGGATG |
| Vim | Forward: CTCCTACGATTCACAGCCA |
|  | Reverse: GAGCCACCGAACATCCT |
| Col1a1 | Forward: CAGAGGCGAAGGCAACA |
|  | Reverse: GTCCAAGGGAGCCACATC |
| Col3a1 | Forward: AGAACCTGGCCGAGATG |
|  | Reverse: TGGACTTCCGGGCATAC |
| Kcnd1 | Forward: TGGGAACGTAGGTAGGGA |
|  | Reverse: GCAAGAGGCCAACTAGGA |
| Kcnd3 | Forward: GGAAAGTACCTGGGAGCA |
|  | Reverse: GAAGTGAGGGAGCCTGAA |
| Scn5a | Forward: AAGGGGAGGGTTGTGGTT |
|  | Reverse: GCCCCAGTGTGTGAAGTG |
| Kcnn3 | Forward: GGGTGGTATTAGGTTTGGG |
|  | Reverse: GGGCGGCTCTCTTTTATT |
| miR-499-5p | Forward: GCGCGTTAAGACTTGCAGTG |
|  | Reverse: AGTGCAGGGTCCGAGGTATT |
|  | RT: GTCGTATCCAGTGCAGGGTCCGAG GTATTCGCACTGGATACGACAAACAT |
| miR-374c-3p | Forward: CGCGCGACTTAGCAGGTTG |
|  | Reverse: AGTGCAGGGTCCGAGGTATT |
|  | RT: GTCGTATCCAGTGCAGGGTCCGAG GTATTCGCACTGGATACGACATAATA |
| miR-29b-1-5p | Forward: GCGGCTGGTTTCATATGGT |
|  | Reverse: AGTGCAGGGTCCGAGGTATT |
|  | RT: GTCGTATCCAGTGCAGGGTCCGAG GTATTCGCACTGGATACGACTAAACC |
